# A Longitudinal Study on Parent-Infant Rest-Activity Rhythms Using Actigraphy From Late Pregnancy to Eight Months After Birth

**DOI:** 10.7759/cureus.76029

**Published:** 2024-12-19

**Authors:** Yoko Asaka, Chie Kondo

**Affiliations:** 1 Nursing, Mie University School of Medicine, Tsu, JPN; 2 Nursing, Tokyo Women's Medical University, Tokyo, JPN

**Keywords:** acceleration sensor, activity rhythms, circadian rhythms, longitudinal study, parent-child

## Abstract

Circadian rhythms develop from an ultradian to a circadian rhythm during a few months in the early human life stage. One of the strong factors in promoting the development of circadian rhythms during infancy is maternal rest-activity rhythms. However, few studies have examined comparing the rest-activity rhythms of parents and infants. This study aimed to describe longitudinal changes in the rest-activity rhythms of a family at seven time points, from late pregnancy to eight months after birth.

Data on a seven-day activity was obtained from Actigraphs (Micro-mini-RC, Ambulatory Monitoring Inc., NY, USA) and analyzing activity data using Action-W4 software (ver. 3.10.0.3, Ambulatory Monitoring Inc.). Double plot diagrams based on the three individuals' average activity levels were created and visually observed longitudinal changes. Also, periodic parameters, midline estimated rhythm (mesor), amplitude, and 24-hour autocorrelation coefficient, were calculated. This study was approved by the Ethics Committee (approval number: U2024-007).

The participants were a mother (38-year-old primipara), a father (34 years old), and a male infant. The male infant was delivered vaginally at 40 gestation weeks (GW). The mother and newborn were discharged from the hospital on day 6 after birth and slept in the same room with the father. Both parents took childcare leave; one year for the mother and one month for the father, respectively. The mesor and the amplitude were both lowest at one month after birth and increased with age. When the difference in the activity counts between active and inactive periods increases, the rest-activity rhythm becomes clearer. The mesor and amplitude, the parameters indicating these differences, show the rest-activity rhythms changing robustly. The 24-hour autocorrelation showed distinctive trends for the father and dyad of mother and infant. The father's value was 0.4-0.6, maintaining regularity of the rest-activity rhythm. The mother’s value decreased to 0.3 at 1 week after birth, approaching the value of the infant, and then the changes gradually approached the father’s value throughout the study period. Also, the values of the mother and child were synchronized, and the mother's value was 0.1-0.3 higher than the child's value at each measurement point.

The regularity of rhythm as indicated by the mesor, amplitude, and 24-hour autocorrelation value reached its lowest value one week after birth among the parents and infant, which means that the periodicity of the rhythm was impaired. One month later, the regularity of the rhythm increased, and the father maintained a high value. The changes in the values ​​of the mother and infant were synchronized, with the mother's value always remaining high. It is possible that the regularity of the father's rhythm contributed to the establishment of a rest-activity rhythm in the mother and infant.

## Introduction

A biological rhythm in humans develops as they are growing. The biological clock is located in the suprachiasmatic nucleus (SCN) and controls the biological rhythm. Among biological rhythms, the rest-activity (sleep-wake) rhythm can be identified relatively early in human life [1]. The rest-activity (sleep-wake) rhythm in the newborn period is characterized as the ultradian rhythm, which is a short sleep-wake cycle of less than 12 hours. The circadian rhythm, synchronizing the 24-hour cycle, is established as early as 2 months after birth to 4 months [2,3]. The most important factor in synchronizing biological rhythms to a 24-hour cycle is the light condition [1], as well as the rearing environment, such as maternal rest-activity rhythm, and feeding conditions [4,5]. It is important to adjust the environment that gives newborns and infants clues about the regular rhythm of day and night from as early an age as possible [5]. In recent years, due to the trend toward nuclear families [6] and the increasing age at which women give birth, couples are increasingly sharing housework and childcare responsibilities in Japan [7]. In other words, fathers are increasingly being involved in child-rearing. Research on the influence of fathers on the development of children's rest-activity rhythms has been limited to studies [8]. We were able to obtain cooperation from one family for a longitudinal survey of activity rhythms. Here, we report on the results of the father's rest-activity rhythms in relation to those of the mother and infant, using an acceleration sensor. This study aims to describe longitudinal changes in the rest-activity rhythms of one family at seven time points, from late pregnancy to eight months after birth.

## Materials and methods

The case family is a nuclear family. The mother was a 38-year-old primipara and the father was 34 years old and both of them were employed and had daytime jobs. The mother took maternity leave from 34 gestational weeks (GW) and took childcare leave for 1 year after the birth. The father also took childcare leave for 1 month after the birth. The father and mother have no medical history. The mother had no complications during pregnancy or delivery. The male infant was delivered vaginally at 40 GW. The mother and newborn were discharged from the hospital on the sixth day after birth and since then, both parents and infant slept in the same room. Regarding childcare duties, the father was responsible for bathing the infant and soothing the infant when he would not sleep at night. The infant was exclusively breastfed from birth through the study period. Weaning food was started at six months of age. The author (KC) recruited the family and analyzed activity data. Both authors evaluated activity rhythm based on the family's activity data and the author (KC) provided feedback to the family.

Data collection

An activity of seven-day data for a pair of parents and an infant were collected at seven measurement points: 33 GW, 1 week after birth, 1 month after birth, 2 months after birth, 3 months after birth, 4 months after birth, and 8 months after birth. The data collection period was from September 2022 to May 2023. The case family was instructed to attach Actigraphs (Micro-mini RC, Ambulatory Monitoring Inc., NY, USA) to their non-dominant arm for the parent and infant’s ankle with an adjustable elastic belt for seven consecutive days at each measurement point and to remove it when there was a possibility of it being exposed to water such as when bathing [9]. Also, the parent of the family was instructed to keep a sleep diary during each data collection period [10]. The actigraphs were set in 1-min epochs in the zero-crossing mode, which is recommended for adequate circadian activity rhythm analysis [9]. 

**Figure 1:**
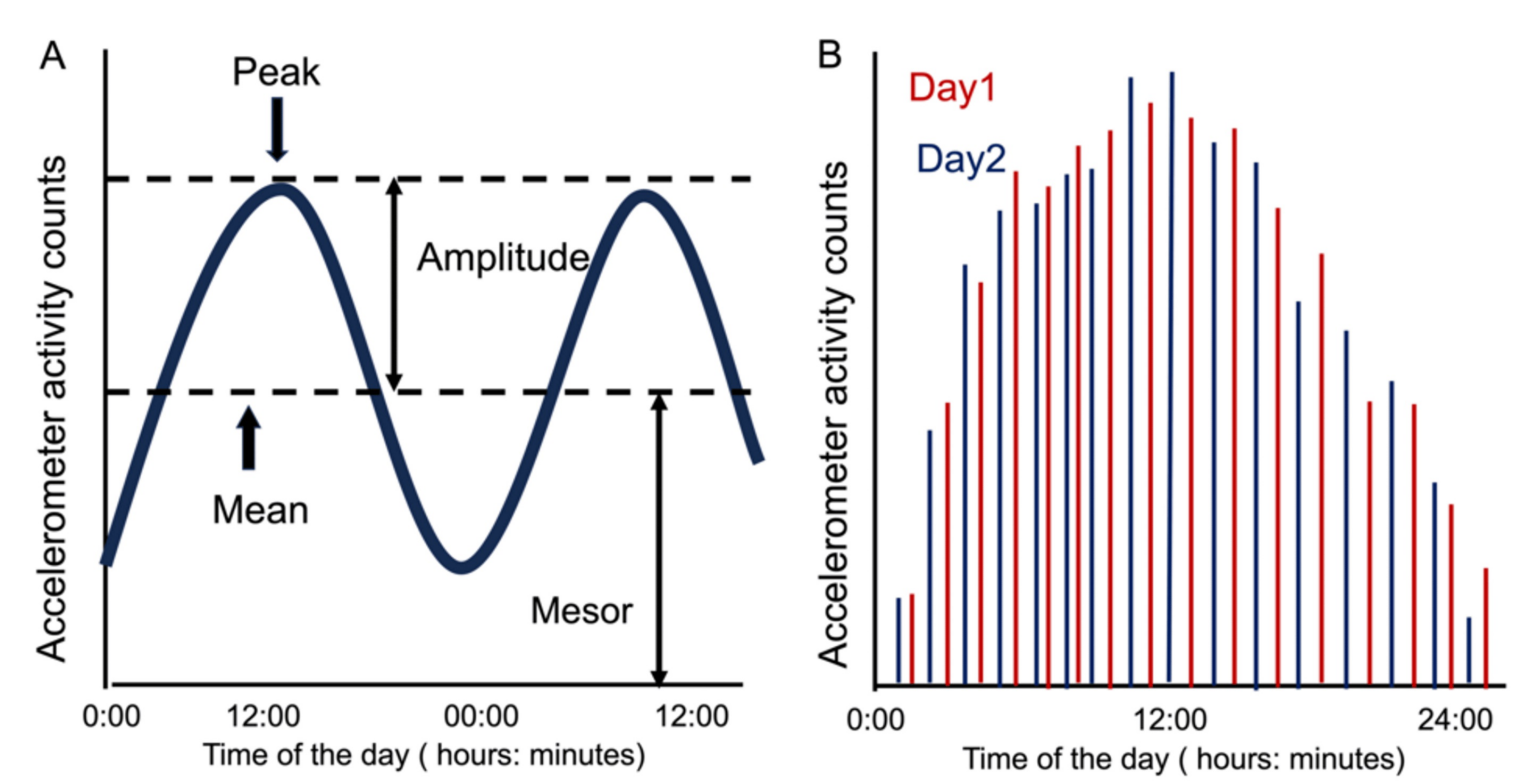
Overview of the 24-hour period analysis of rest-activity using the cosinor method A: explanation of mesor and amplitude, B: explanation of 24-h autocorrelation 1A shows an explanation of mesor and amplitude used to evaluate rhythm amplitude. By finding the cosine curve that best matches the measured activity count, the parameters mesor, amplitude, and acrophase are calculated. In this study, mesor and amplitude, which indicate rhythm amplitude, were used as parameters to capture changes in rest-activity rhythm formation. 1B shows the overlap of activity count on two different days when the actigraph was worn. In this example, for convenience, only the first and second days are shown as fictitious data. The 24-hour autocorrelation coefficient is defined as a comparison of the regularity and consistency of the rhythm from one day to the next. Therefore, the higher the 24-hour autocorrelation coefficient, the higher the correlation between the rest-activity time distributions on days 1 and 2, which can be interpreted to mean that there is regularity in the daily rest-activity rhythm.

Each completed data-collection activity data recorded by the Actigraph was downloaded using ActMe software (ver. 3.10.0.3, Ambulatory Monitoring Inc.), and then double plot diagrams based on the three individuals' average activity levels were created and visually observed longitudinal changes. Next, the periodic parameters, midline estimated rhythm (mesor), amplitude, and 24-hour autocorrelation [11,12] were calculated and analyzed for rest-activity rhythms. Mesor is defined as the 24-hour rhythm-adjusted mean of the activity counts and higher values represent a more robust activity. Amplitude is defined as the peak value of the cosine curve minus the mesor and the value represents the rhythmic change of an individual’s activity during the 24 hours. The 24-hour autocorrelation coefficient is defined as a comparison of the regularity and consistency of the rhythm from one day to the next. The value ranges from -1.0 to +1.0 and the optimal value is +1.0. The overview of these parameters is explained in Figure 1.

This study was conducted with the approval of the Institutional Review Board of the Clinical Research Ethics Review Committee of Mie University Hospital (Ethics Number: U2024-007).

## Results

**Figure 2:**
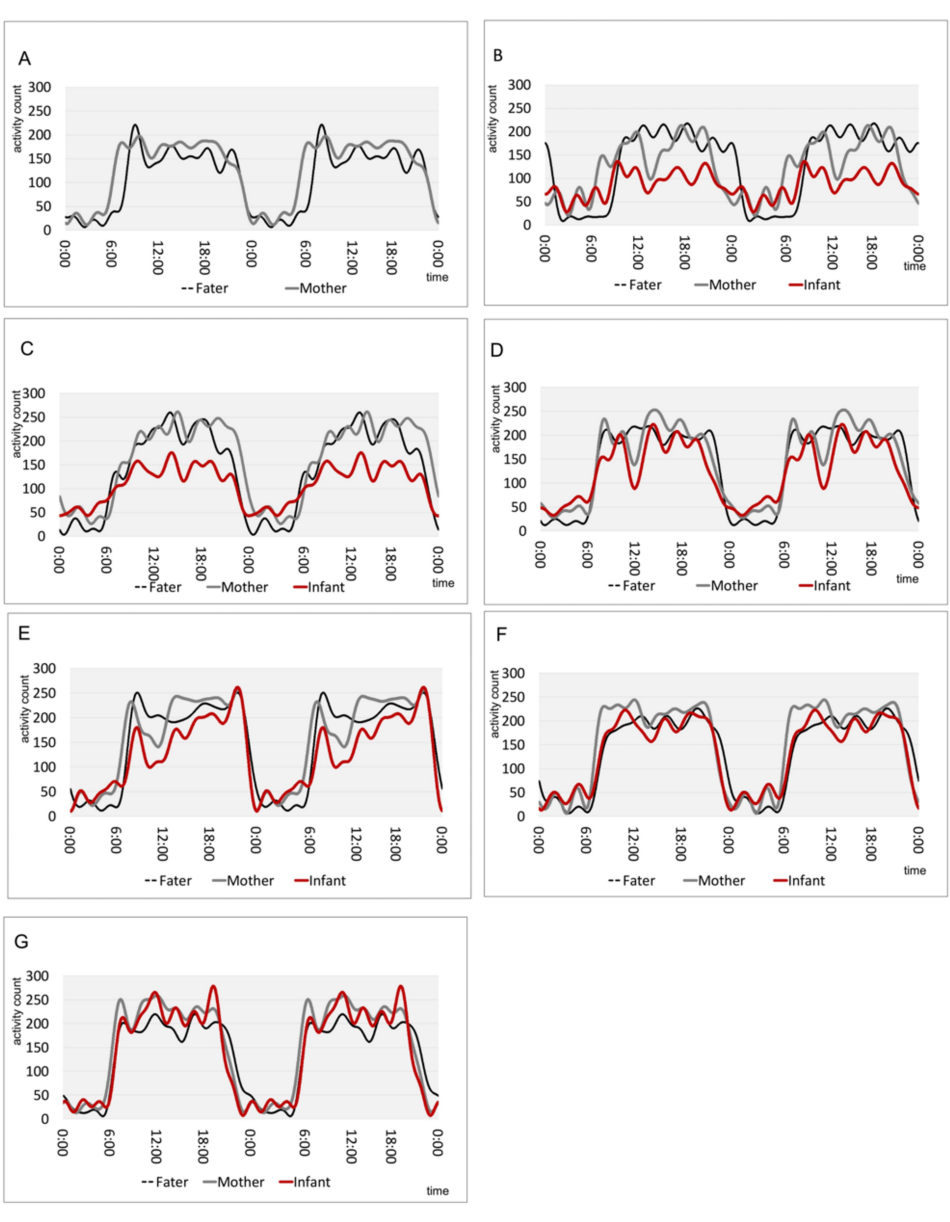
Double plot diagram of rest-activity patterns of father, mother, and infant at each measurement point A: 33 gestational weeks, B: 1 week after birth, C: 1 month after birth, D: 2 months after birth, E: 3 months after birth, F: 4 months after birth, G: 8 months after birth The triplet double plots at the measurement points were arranged. In the double plot diagram, the vertical axis represents average activity and the horizontal axis represents time. The time starts at midnight. Generally, there is a tendency for activity to be low at night and high during the day.

The average activity over 24 hours for seven points is overviewed in a double-plot diagram (Figure 2). At 33 weeks of pregnancy, both father and mother showed a clear biphasic pattern of high daytime activity and low nighttime activity (Figure 2A). One week after birth, the mother's double plot changes like that of the infant, and it contains short-term periodic components (Figure 2B). At one month of age, the double plot diagram of the infant depicts two-layered elements, and the three overlapping plot diagrams are similar (Figure 2C). At two months of age, the double plots of the infants show that the average activity of the infants is increasing and approaching that of the parents (Figure 2D). At four months of age, the infant's double plots showed that the activity level was lower than that of the parents, but the activity rhythm approached that of the parents (Figure 2E), and at eight months of age, the infant's double plots showed that both activity level and activity rhythm were consistent with those of the parents (Figure 2F). The double plots of the father show a clear biphasic pattern, and the double plots of the mother are similar to those of the infants. This trend continues until the age of eight months.

**Figure 3:**
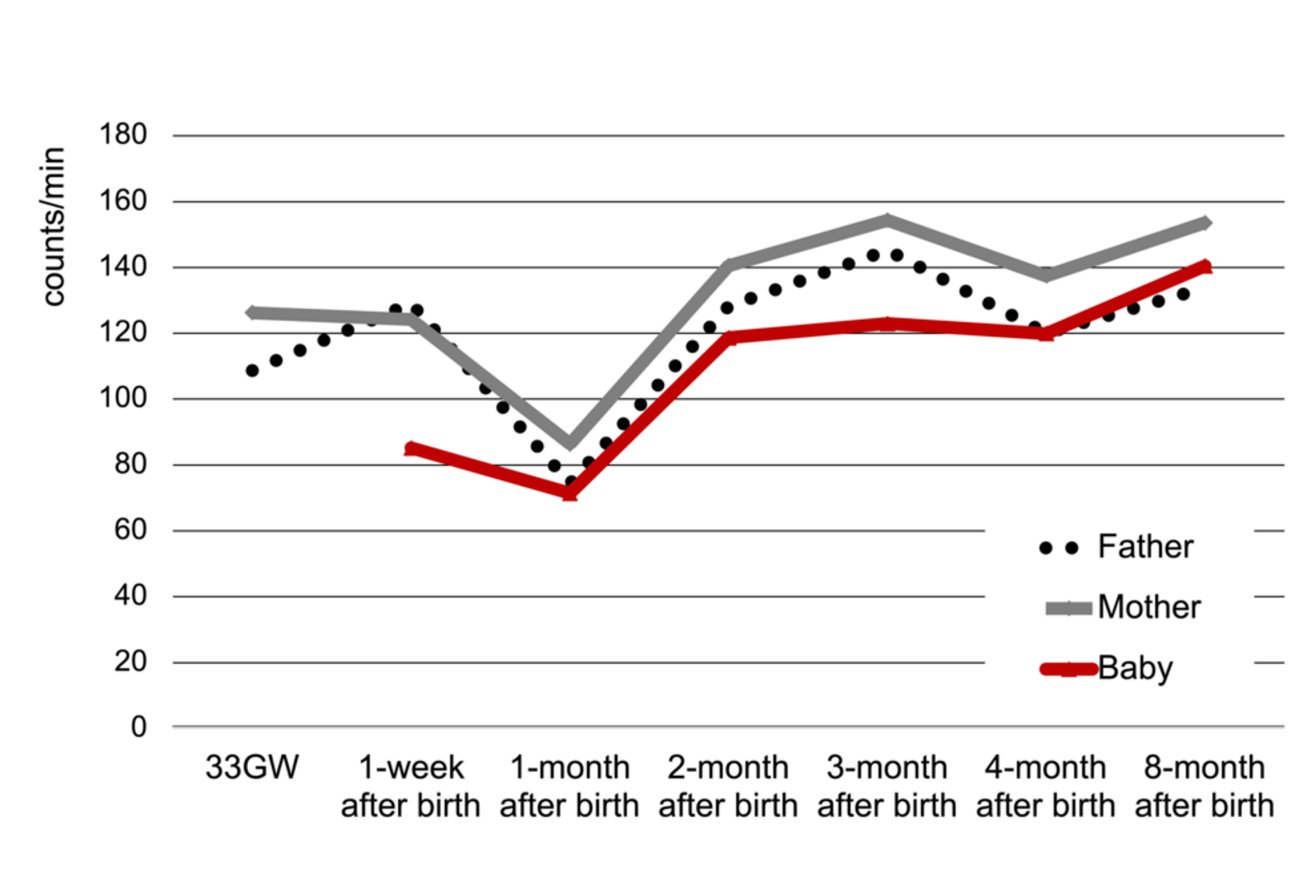
Longitudinal changes of father-mother-infant in mesor This figure shows the midline estimated rhythm (mesor) at each measurement time point for the father, mother, and infant. The vertical axis is mesor (count/min); high values are interpreted as robust activity. The horizontal axis is the measurement period, which is the 7 measurement points, starting from 33 weeks of pregnancy and ending 8 months after birth.

The mesor (counts/min) of the father decreased from 108.7 at 33 GW and 129.0 at 1 week to 74.4 at 1 month after birth and increased to 128.3 at 2 months, and remained during 3 to 8 months (3 months; 145.2, 4 months 120.0, and 8 months 133.8). The value of the mother also decreased from 126.3 at 33 GW and 124.2 at 1 week to 86.4 at 1 month after birth, increased to 140.6 at 2 months, and remained during 3 to 8 months (3 months 154.3, 4 months 137.5, and 8 months 153.6). The value of the infant was 85.2 at 1 week, 71.4 at 1 month, 118.6 at 2 months, 130.0 at 3 months, 119.9 at 4 months, and 140.5 at 8 months (Figure 3).

**Figure 4:**
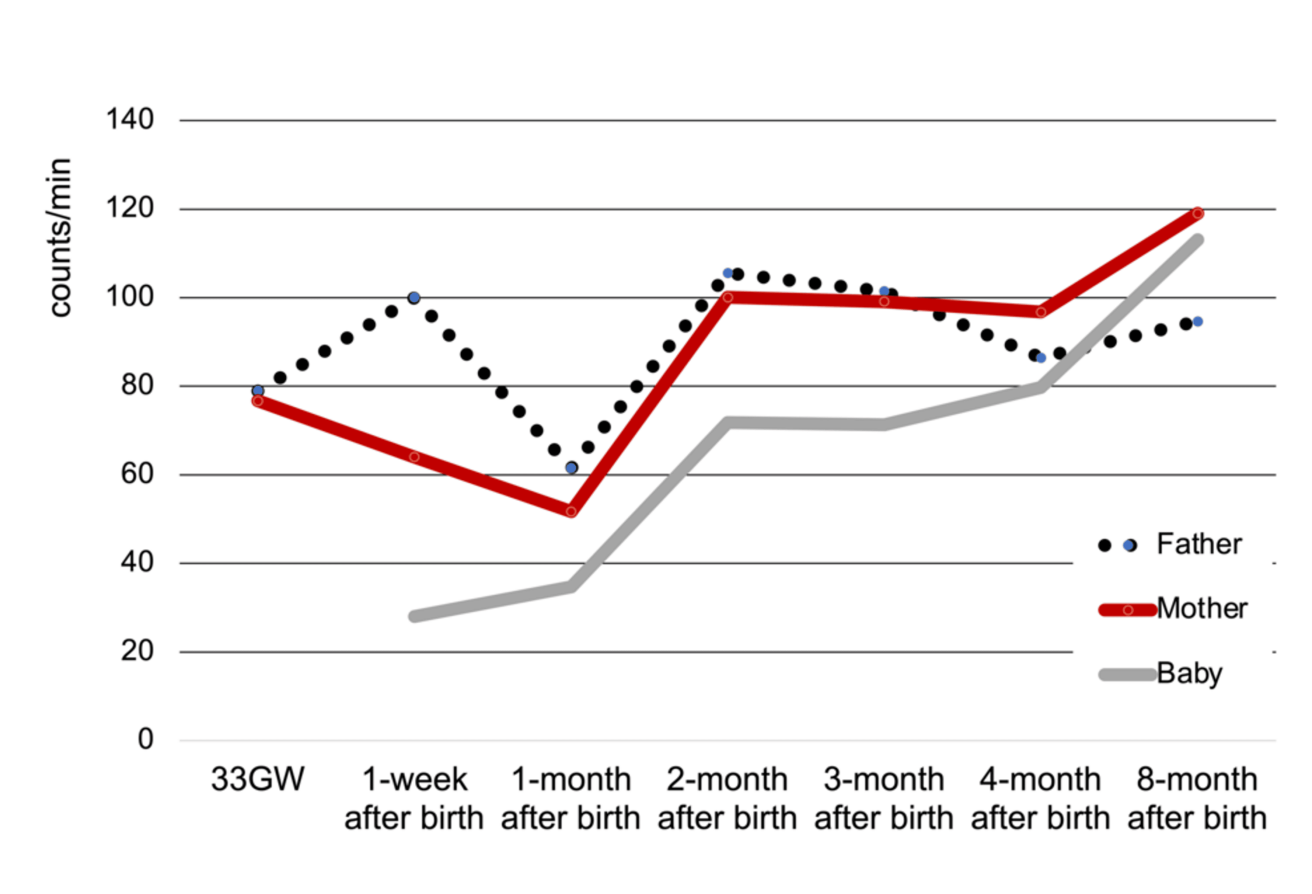
Longitudinal changes of father-mother-infant in amplitude This figure shows the amplitude at each measurement time point for the father, mother, and infant. The vertical axis is amplitude (count/min); high values are interpreted as robust activity. The horizontal axis is the measurement period, which is the 7 measurement points starting from 33 weeks of pregnancy and ending 8 months after birth.

Amplitude (counts/min) of the father was 79.0 at 33 GW, 100 at one week after birth, and dropped to 61.5 at 1 month after birth, and increased to 105.6 at 2 months at peak, then maintained above 80 (3 months 101.5, 4 months 86.4, 8 months 94.7). The value of the mother was 76.7 at 33 GW and decreased to 64.0 at 1 week and 51.5 at 1 month after birth, and increased to 100 at 2 months, and remained during 3 to 4 months (3 months 99.1, 4 months 96.8,) and increased to 119.0 at 8 months. The value of the infant was 28.0 at one week after birth, 34.8 at one month, 71.9 at two months, 71.3 at 3 months, 79.8 at 4 months, and 113.1 at 8 months (Figure 4).

**Figure 5:**
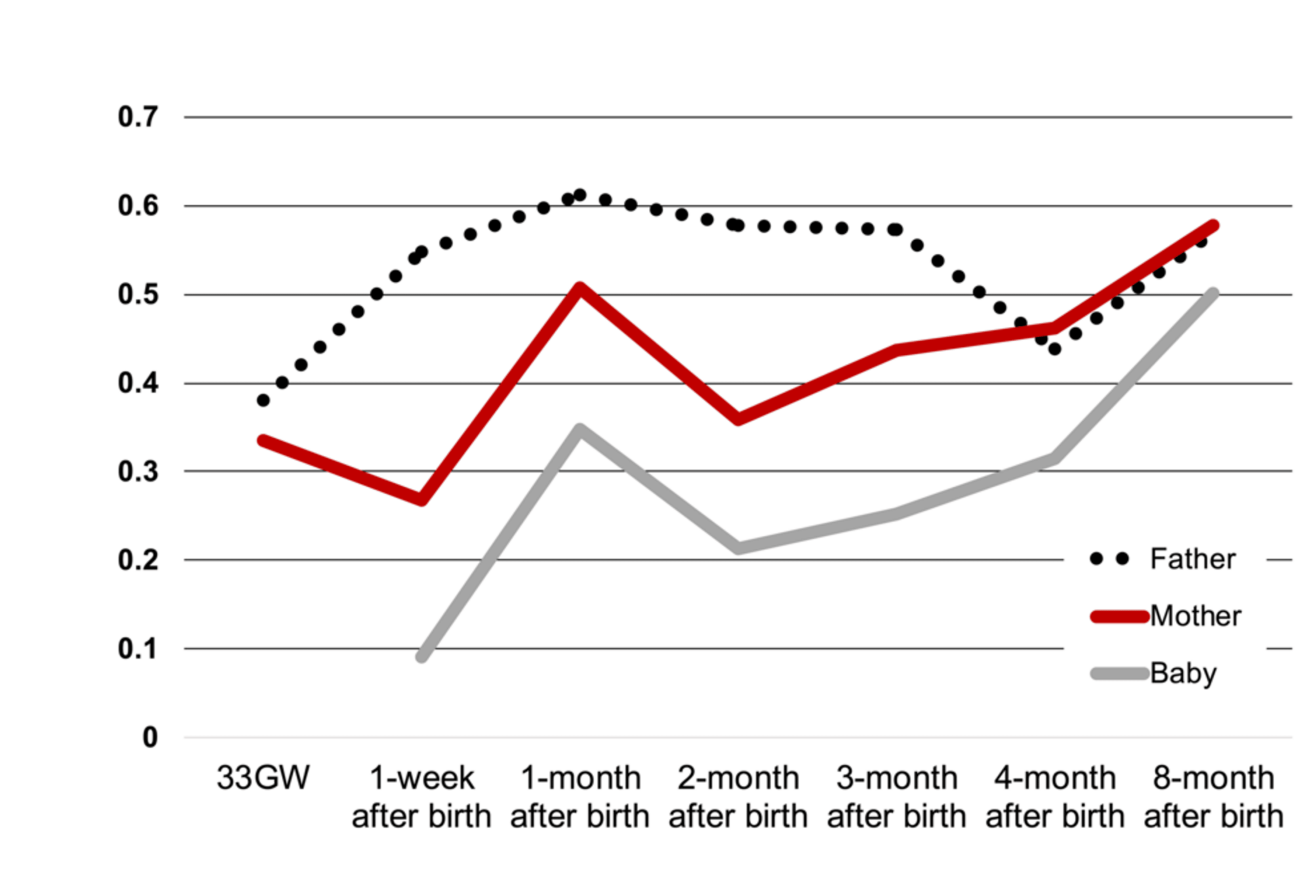
Longitudinal changes of father-mother-infant in the 24-hour autocorrelation coefficient It shows the 24-hour autocorrelation at each measurement time point for the father, mother, and infant. The vertical axis is the coefficient of 24-hour autocorrelation, ranging from -1.0 to +1.0, and the closer to 1, the more regular the rhythm is. The horizontal axis is the measurement period, which is the 7 measurement points starting from 33 weeks of pregnancy and ending 8 months after birth.

In the 24-hour autocorrelation coefficient, the fathers' value was 0.38 at 33 GW; afterward, it maintained at 0.6 until 8 months after birth, except at 4 months (1 week 0.55, 1 month 0.61, 2 months 0.58, 3 months 0.58, 4 months 0.44, 8 months 0.57). Values of mother and infant showed similar changes, but then the values were 0.1 to 0.3 higher for mothers (33 GW 0.34, 1-week mother/infant were 0.27/0.09 at 1 week after birth, 0.51/0.35 at 1 month, 0.36/0.21 at 2 months, 0.43/0.26 at 3 months, 0.46/0.32 at 4 months, and 0.58/0.50 at 8 months (Figure 5).

## Discussion

We described one pair of parents and an infant in a nuclear family and circadian rhythm changes based on activity data longitudinally and analyzed the data by periodic analysis.

Observing the double plot of the average activity rhythm, it was confirmed that the father's activity rhythm was multilayered during the first week and first month of childcare leave, but the biphasic pattern was clearer compared to the mother's. In addition, after returning to work after childcare leave, the pattern returned to a biphasic pattern, and the maternal and child activity rhythms tended to approach the father's biphasic pattern. Previous studies have reported that the daily life of fathers is not affected by the birth of a child [8]. However, this study showed that the activity rhythms of fathers who take parental leave and raise their children together with their mothers are closer to the daily rhythms of infants, including their short cycles.

This progress could also be shown numerically by the results of the parameters of periodic analysis. Mesor decreased in both parents one month after the birth, approaching the infant's level in both parents. Then, at two months after birth, the child's level increased and the parent's level also recovered. Mesor was reported to be 138.2 to 150.3 (counts/min) in healthy adults [11,13]. Amplitude showed a similar change to mesor and was reported 109.0 to 112.4 (counts/min) in a healthy adult [11,13]. For these two parameters, high values ​​are interpreted as robust activity. Therefore, it became clear that at two months of age, the parents' activity levels change to approach their natural levels, and that the infants' levels also change to approach those of his parents.

The trends in the 24-hour autocorrelation coefficient were different between the father and the dyad of the mother and infant. The father's lowest value was at 33 GW, after which it improved and remained consistently high. The mother's value was synchronized with the infant's progress, with the lowest value occurring one week after birth and improving from one month. Autocorrelation is an index that expresses the correlation between shifted data and original data [14]. Therefore, the circadian rhythm of the mother and infant was at its weakest in the first week after birth and increased at one month after giving birth, which means that a daily rhythm was formulated during the first month. Previous literature has shown that breastfeeding promotes the circadian rhythm of infant activity [15] while other papers report that breastfed infants cry more and sleep less than those formula-fed [16,17]. In this study, by tracking the 24-hour regularity of mother and infant during exclusive breastfeeding, we were able to show that the rhythms of mother and infant are similar and that the formation of a 24-hour rhythm progresses.

The results of this study were consistent with that of a previous study [5], which reported the influence of mothers on the formation of an infant's circadian rhythms was confirmed. Thomas et al. described longitudinal rest-activity patterns of the dyad of mother and infant from birth to 12 weeks after the birth and reported development of the circadian rhythm of infants was driven by the mother's 24-hour rhythm, suggesting the importance of maintaining the mother’s 24- hour rest-activity rhythms [5]. Furthermore, the result of this study showed that the regular lifestyle indicated by the father's 24-hour autocorrelation may have inhibited the mother's 24-hour autocorrelation from decreasing, which in turn may have contributed to the formation of the child's circadian rhythm. Although bedtime routine during childhood was proposed in previous studies and one of the related factors is the family factor [18], there are scars about examining the role of family members except the mother in acquiring circadian rhythms. We believe that we have demonstrated the need to verify that the family's daily rhythm contributes to the formation of children's rhythms as a synchronizing factor.

This is a case report, presenting the longitudinal results of a single family. The results of rest-activity rhythms of father-mother-infant using actigraphy are valuable. However, the presenting results from a single family are also a limitation of this study. The influence of the father's rest-activity rhythms on those of the mother and infant is thought to be influenced by various factors such as the parents' age, occupation, length of childcare leave, degree of involvement in childcare, sleeping environment, and marital relationship [18]. Therefore, it is necessary to increase the number of cases, conduct multiple analyses on a case-by-case basis, and add statistical analysis.

## Conclusions

The rhythm regularity, as indicated by mesor, amplitudes, and 24-hour autocorrelation values, reached its lowest value between parents and infants one week after birth, implying that the rhythm periodicity of the parents was impaired. After one month, rhythm regularity increased, with the father maintaining higher values. Changes in values ​​for the mother and infant were synchronous, with the mother's values ​​always remaining higher than those of the infant. It is possible that the regularity of the father's rhythm contributed to the maintenance of the mother's rhythm periodicity, which in turn contributed to the establishment of the infant's rest-activity rhythm. Sleep habits are developed by establishing a 24-hour sleep-wake cycle. Therefore, the possibility that the father's regular rest-activity may encourage the child's rest-activity in the early stages of life leads to the adjustment of the child's sleep habits.
